# Analysis of the effectiveness and efficiency of the Indonesian metastatic bone disease of unknown origin algorithm (INA-MBD): time to diagnosis and cost to diagnosis: Quasi-experimental study

**DOI:** 10.12688/f1000research.146118.1

**Published:** 2024-04-23

**Authors:** Yuni Artha Prabowo Putro, Teguh Aryandono, Irianiwati Widodo, Rahadyan Magetsari, Dibyo Pramono, Muhammad Phetrus Johan, Moh Asri Abidin, Ardanariswara Wikantyasa, A Faiz Huwaidi, Paramita Ayu Saraswati

**Affiliations:** 1 Doctoral Program in Medicine and Health Sciences, Faculty of Medicine, Public Health and Nursing, Universitas Gadjah Mada, Yogyakarta, D.I. Yogyakarta, 55281, Indonesia; 2 Orthopedics and Traumatology, RSUP Dr. Sardjito Hospital, Jl. Kesehatan Sendowo, , Sleman, D.I. Yogyakarta, 55281, Indonesia; 3 Faculty of Dentistry, Universitas Gadjah Mada, Yogyakarta, D.I. Yogyakarta, 55281, Indonesia; 4 Orthopaedic and Traumatology, RSUP Dr. Wahidin Sudirohusodo, Sulawesi Selatan, 90245, Indonesia; 5 Faculty of Medicine, Universitas Hasanuddin, Makassar, Sulawesi Selatan, 90245, Indonesia; 6 Faculty of Medicine and Health Sciences, Universitas Muhammadiyah Makassar, Makassar, Sulawesi Selatan, 90221, Indonesia

**Keywords:** metastatic bone disease, Neoplasm, unknown primary, algorithm, management, cost effectiveness, diagnosis

## Abstract

**Background:**

Patients with Metastatic Bone Disease (MBD) often presenting with complaints of pain and multiple osteolytic lesions findings. Remarkably, 30% of these cases exhibit an undetected primary lesion. Hence, categorizing them as MBD of unknown origin. The diagnostic processes of patients with MBD of unknown origin typically takes up to four months, rendering it as a catastrophic disease with the second-highest financial burden. Given its urgency, it is necessary to develop a systematic and evidence-based consensus for managing cases of MBD with an unknown origin.

**Purpose:**

This study aimed to enhance the effectiveness and efficiency of treating patients with MBD of unknown origin through the application of the INA-MBD algorithm.

**Research method:**

A quasi-experimental study with a pretest and post-test design was conducted with a total of 128 patients who met the inclusion and exclusion criteria. The patients were consecutively enrolled and categorized into two groups: the intervention group with the INA-MBD algorithm and the non-intervention group without the INA-MBD algorithm. The primary outcomes were the cost and time to diagnose MBD of unknown origin. The proposed measuring tool was the INA-MBD algorithm. Furthermore, for the cost-to-diagnosis variable, an extra measurement tool was used, which were summaries of the patient’s medical bill including hospital stays and medical procedures. The analysis of data related to the time-to-diagnosis variable was conducted using the Log Rank regression test, and cost-to-diagnosis variable was carried out using co-variance test.

## Introduction

Patients diagnosed with Metastatic Bone Disease (MBD) of unknown origin are at greater risk of higher mortality and morbidity rates, mainly due to skeletal-related events, with a solely 5% survival rate over a 5-year period. ^1^ In three-quarters of MBD cases, the diagnosis of primary malignancy is made after the patient’s condition deteriorates, typically around four months into the progression of the disease. ^2^ Alarmingly, it has been brought to attention that late establishment of the primary cancer diagnosis significantly contributes to treatment delay up to 86%. ^3^ The prolonged process of diagnosing the unknown origin of MBD is further exacerbated by a series of routine laboratory examinations, including tumor markers. ^4^

Prognosis of patients with MBD of unknown origin highly relies on the timing of diagnosis and treatment. There is no current guideline on the selection of preliminary examinations to identify primary lesions in MBD. ^1^ This lack of consensus adds to the financial burden, with Indonesian national health insurance allocating 18% of the total budget for catastrophic diseases to the care of cancer patients. ^5^ In the United States, the direct cost of MBD is approximately $75,329, double the cost of treating cancer patients without MBD, at $31,382 per year. ^6^^,^^7^ Common diagnostic procedures involve a series of laboratory tests such as tumor markers and ALP, X-rays of the chest and affected limbs, CT scans, MRIs, bone scans, and PET scans, followed by bone or organ biopsies. These procedures contribute to prolonged diagnosis times and increased financial burdens, intensifying the complexities associated with addressing MBD-related challenges.

Certainly, we have developed the INA-MBD algorithm based on our literature review and clinical experience in managing patients with MBD of unknown origin. This algorithm provides tailored recommendations for prioritizing supporting examinations based on the patient’s clinical condition. Diverging from conventional methods, the screening checks in the INA-MBD algorithm are not executed sequentially and exhaustively. The main objective is for an early and secure biopsy to speed up the diagnostic process, leading to reduced further examination costs without compromising the success of diagnosing MBD of unknown origin.

**Objective**: The objective of this study was to assess the impact of implementing the INA-MBD algorithm on the direct treatment costs and the time to diagnose primary malignancy in patients with MBD of unknown origin.

## Protocol

### Study design

This research employed a quasi-experimental pre-test and post-test design, categorizing participants into an intervention group and a non-intervention group. The intervention group utilized the INA-MBD algorithm for diagnosing patients with MBD of unknown origin, while the non-intervention group used the intra-hospital conventional diagnosis algorithm.

### Patients and eligibility criteria

In this study, the intervention group consisted of consecutively collected patients from inpatients, outpatients, and emergency departments diagnosed with MBD of unknown origin until reaching the required sample size. The INA-MBD algorithm was applied for the intervention group. For the non-intervention group, medical record data was utilized from MBD-diagnosed patients with unknown primary lesions between 2018 and 2022 at RSUP Dr. Sardjito (Yogyakarta, Indonesia) and Dr. Wahidin Sudirohusodo (South Sulawesi, Indonesia).

### Inclusion criteria

a. Patients with a final diagnosis of MBD
b. Patients without a history of malignancy
c. Patients who agreed to participate in the this study and signed the written informed consent form.

### Exclusion criteria

a. Patients with incomplete medical record data
b. Patients who did not undergo operative procedures.

### Study settings

This multicentre research was conducted at two tertiary referral hospital in Indonesia, RSUP Dr. Sardjito in Yogyakarta and Dr. Wahidin Sudirohusodo in South Sulawesi.

### Data collection, management, and analysis

**Data collection**

Consecutive sampling was employed, including all patients meeting the inclusion criteria until the required sample size was met.

### Variable

**Independent variable**

**Figure 1.**
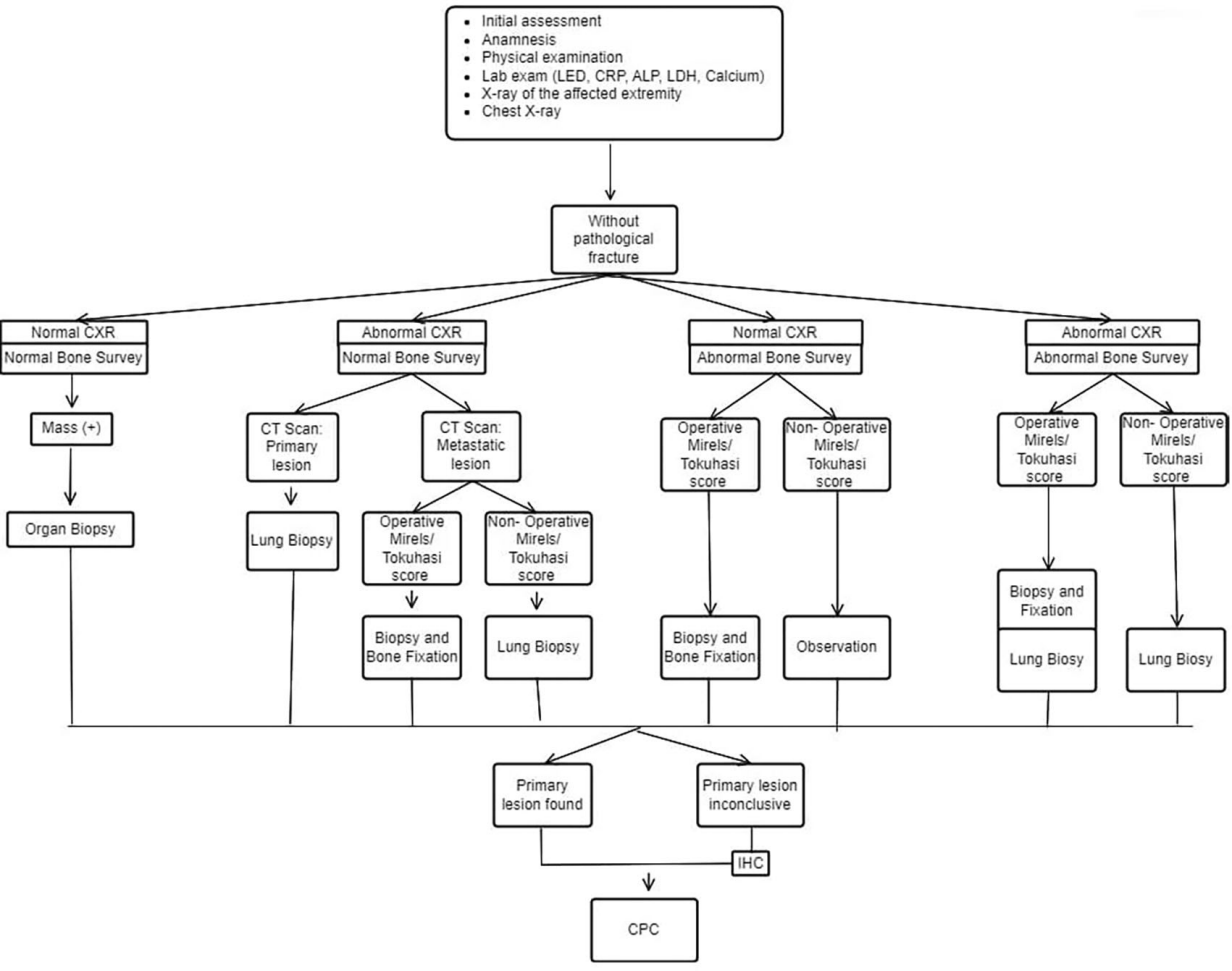
INA-MBD algorithm without pathological fracture. This figure is our original creation synthesized from both literature and our clinical experience. Show steps and list of management in patients with MBD of unknown origin without pathological fracture. CXR (Chest X-Ray), IHC (Immunohistochemistry), CPC (Clinicopathological conference)/ multidisciplinary team discussion

**Figure 2.**
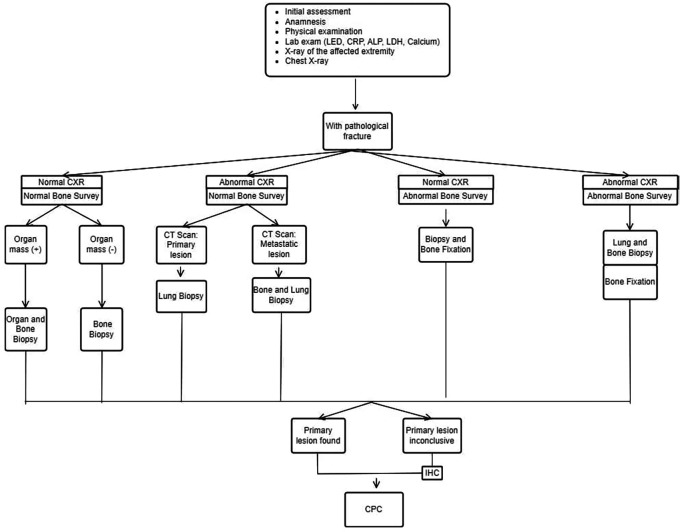
INA-MBD algorithm with pathological fracture. This figure is our original creation synthesized from both literature and our clinical experience. Show steps and list of management in patients with MBD of unknown origin with pathological fracture. CXR (Chest X-Ray), IHC (Immunohistochemistry), CPC (Clinicopathological conference)/multidisciplinary team discussion.

The INA-MBD algorithm ( Figures 1 and 2) will be used as the independent variable. The interventional group will use the INA-MBD algorithm as a management guideline, while the non-interventional group relied on retrospective data from medical records.

### Dependent and confounding variable

The dependent variables were time-to-diagnosis and cost-to-diagnosis. Confounding variables, such as gender, age, type of primary cancer, and metastatic location, were derived from medical records for the non-interventional group and other various sources for the interventional group.

### Data analysis

Bivariate analysis between time to diagnose or cost to diagnose and the use of the INA-MBD algorithm will employ an independent T-test, with Mann-Whitney as an alternative method if the data exhibit abnormal distribution. For the analysis of time to diagnose, the survival rate will be assessed using Kaplan-Meier and log-rank regression tests. To evaluate the hypothesis concerning the cost to diagnose, a covariance test will be employed to analyse the regression mean of both groups.

### Dissemination

After the completion of the study, we will publish it in an indexed journal or conference.

### Study status

The study was in the data collection period and is planned to finish collecting data by December 2024.

### Registration

This research will be registered on Researchregistry.com.

## Discussion

The diagnostic challenge in identifying primary malignancies often leads to patients with MBD of unknown origin being misdiagnosed with primary bone tumors or hematological malignancies. An additional complication is that not all MBD patients have a cancer history, with 71% being diagnosed with their primary malignancy only after a clinical deterioration. ^2^ In addition, therapeutic approaches for MBD necessitate concurrent treatment for the primary malignancy.

The diagnostic process for primary malignancy in MBD typically involves a series of relatively non-invasive imaging examinations. Tsukamoto et al. (2021) advocate for plain X-rays, CT scans, and MRI for all patients with bone lesions. If bone destruction is observed without periosteal reaction, then metastasis is suspected, prompting further examinations like abdominal, thoracic, or pelvic CT scans, tumor markers, serum electrophoresis, Bence Jones protein, and bone scans or PET/CT scans. Biopsy is reserved for the conclusion after completing all imaging and laboratory examinations. ^8^ Tumor marker examinations, including CEA, CA-125, CA19-1, and AFP, exhibit low sensitivity and specificity in determining the primary malignancy in MBD of unknown origin. Tumor markers are not exclusively produced by malignant cells, making serum tumor markers more relevant for therapy monitoring or determining cancer prognosis. ^4^

The extensive use of radiological examinations extends the time required to establish a primary malignancy diagnosis, contributing to an increased financial burden. Therefore, there is a crucial need for selective use of supportive imaging examinations. Imaging examinations of the gastrointestinal and female reproductive organs often result in waste of time and resources. Thus, not recommended as routine examinations in diagnosing primary lesions in MBD of unknown origin. ^1^ Bone scans alone cannot confirm malignancy origin in MBD and still require a bone survey for lesion confirmation. The cost-effectiveness of a bone survey influences our preference for it over a bone scan. ^9^

More invasive examinations, such as biopsy, can confirm the origin of the primary malignancy in MBD patients. Biopsy can be performed on metastatic bone lesions, with or without pathological fractures, and on lesions suspected to be primary malignancies. Bone biopsies can confirm the origin of malignancy up to 70%. ^10^

We have devised an algorithm named INA-MBD through clinical and evidence-based methods. The INA-MBD algorithm offers guidelines for managing patients with MBD of unknown origin, in concordance with the patient’s clinical condition, whether or not pathological fractures are present. This aims to provide more selective guidance in choosing imaging modalities. The INA-MBD algorithm excludes serum tumor marker examinations as a diagnostic tool for the reasons explained above. It also serves as a guide for performing biopsies not only on suspected primary lesions but also on bone lesions. This research seeks to evaluate the effectiveness and efficiency of using the INA-MBD algorithm in diagnosing primary malignancies in patients with MBD of unknown origin. The measurable variables representing the effectiveness of the INA-MBD algorithm include time-to-diagnosis and the cost-effectiveness of managing patients with MBD of unknown origin.

### Ethical considerations

This study has Ethical approval from The Medical and Health Research Ethics Committee (MHREC) Faculty of Medicine, Public Health and Nursing Universitas Gadjah Mada- Dr. Sarjito General Hospital on 09 Jan 2023 and the protocol number is KE/FK/0042/EC/2023 Patients who agreed to participate in the study signed the written consent form.

## Author contributions

Conceptualization: (Y.A.P., A.W., T.A., I., R.M., D.P.) Data Curation: (Y.A.P., D.P., A.W., P.A.S., A.F.) Formal Analysis: (Y.A.P.)., A.W., T.A., I., R.M., D.P) Investigation: (Y.A.P., A.W., P.A.S., A.F) Methodology: (Y.A.P., D.P., A.W., M.P.J., M.A.A.) Software: (Y.A.P., P.A.S., A.F.) Supervision: (T.A., I., RM., D.P., M.P.J., M.A.A.) Validation: (Y.A.P., T.A. I., R.M., D.P., M.P.J., M.A.A.) Writing original draft: (Y.A.P., A.W., P.A.S., A.F.) Writing review & editing: Y.A.P., T.A., I., R.M., D.P., M.P.J., M.A.A., A.W., P.A.S., A.F.).

## Data Availability

No data are associated with this article. Spirit Outcome Checklist is available at *Zenodo: Checklist for” Analysis of the effectiveness and efficiency of the Indonesian metastatic bone disease of unknown origin algorithm (INA-MBD) time to diagnosis and cost to diagnosis: Quasi Experimental Study”*, DOI 10.5281/zenodo.10901730.
