# Analysis of the effectiveness and efficiency of the    Indonesian metastatic bone disease of unknown origin algorithm (INA-MBD): time to diagnosis and cost to diagnosis : Quasi-experimental study

**DOI:** 10.12688/f1000research.146118.2

**Published:** 2024-07-09

**Authors:** Yuni Artha Prabowo Putro, Teguh Aryandono, Irianiwati Widodo, Rahadyan Magetsari, Dibyo Pramono, Muhammad Phetrus Johan, Moh Asri Abidin, Ardanariswara Wikantyasa, A Faiz Huwaidi, Paramita Ayu Saraswati

**Affiliations:** 1 Doctoral Program in Medicine and Health Sciences, Faculty of Medicine, Public Health and Nursing, Universitas Gadjah Mada, Yogyakarta, D.I. Yogyakarta, 55281, Indonesia; 2 Orthopedics and Traumatology, RSUP Dr. Sardjito Hospital, Jl. Kesehatan Sendowo, , Sleman, D.I. Yogyakarta, 55281, Indonesia; 3 Faculty of Dentistry, Universitas Gadjah Mada, Yogyakarta, D.I. Yogyakarta, 55281, Indonesia; 4 Orthopaedic and Traumatology, RSUP Dr. Wahidin Sudirohusodo, Sulawesi Selatan, 90245, Indonesia; 5 Faculty of Medicine, Universitas Hasanuddin, Makassar, Sulawesi Selatan, 90245, Indonesia; 6 Faculty of Medicine and Health Sciences, Universitas Muhammadiyah Makassar, Makassar, Sulawesi Selatan, 90221, Indonesia

**Keywords:** metastatic bone disease, Neoplasm, unknown primary, algorithm, management, cost effectiveness, diagnosis

## Abstract

**Background:**

Patients with Metastatic Bone Disease (MBD) often present with complaints of pain and multiple osteolytic lesions findings. Remarkably, 30% of these cases exhibit an undetected primary lesion. Hence, categorizing them as MBD of unknown origin. The diagnostic processes of patients with MBD of unknown origin typically takes up to four months, rendering it as a catastrophic disease with the second-highest financial burden. Given its urgency, it is necessary to develop a evidence-based consensus for managing cases of MBD with an unknown origin.

**Purpose:**

This study aimed to enhance the effectiveness and efficiency of treating patients with MBD of unknown origin through the application of the INA-MBD algorithm.

**Research method:**

A quasi-experimental study with a pretest and post-test design was conducted with a total of 128 patients who met the inclusion and exclusion criteria. The patients were consecutively enrolled and categorized into two groups: the intervention group with the INA-MBD algorithm and the non-intervention group without the INA-MBD algorithm. The primary outcomes were the cost and time to diagnose MBD of unknown origin. The proposed measuring tool was the INA-MBD algorithm. Furthermore, for the cost-to-diagnosis variable, an extra measurement tool was used, which were summaries of the patient’s medical bill including hospital stays and medical procedures. The analysis of data related to the time-to-diagnosis variable was conducted using the Log Rank regression test, and cost-to-diagnosis variable was carried out using co-variance test.

## Introduction

Patients diagnosed with Metastatic Bone Disease (MBD) of unknown origin are at greater risk of higher mortality and morbidity rates, mainly due to skeletal-related events, with a solely 5% survival rate over 5 years. ^1^ In three-quarters of MBD cases, the diagnosis of primary malignancy is made after the patient's condition deteriorates, typically around four months into the progression of the disease. ^2^ Alarmingly, it has been brought to attention that late establishment of the primary cancer diagnosis significantly contributes to treatment delay up to 86%. ^3^ The prolonged process of diagnosing the unknown origin of MBD is further exacerbated by a series of routine laboratory examinations, including tumor markers. ^4^

The prognosis of patients with MBD of unknown origin highly relies on the timing of diagnosis and treatment. The primary challenge in managing patients with MBD of unknown origin lies in the absence of consensus on prioritizing supporting examinations. Unfortunately, there is no current guideline on the selection of preliminary examinations to identify primary lesions in MBD. ^1^ This lack of consensus adds to the financial burden, with Indonesian national health insurance allocating 18% of the total budget for catastrophic diseases to the care of cancer patients. ^5^ In the United States, the direct cost of MBD is approximately $75,329, double the cost of treating cancer patients without MBD, at $31,382 per year. ^6^^,^^7^ Common diagnostic procedures involve a series of laboratory tests such as tumor markers and ALP, X-rays of the chest and affected limbs, CT scans, MRIs, bone scans, and PET scans, followed by bone or organ biopsies. These procedures contribute to prolonged diagnosis times and increased financial burdens, intensifying the complexities associated with addressing MBD-related challenges.

Certainly, we have developed the INA-MBD algorithm through a rigorous scientific process. We conducted a systematic review, registered in the research registry with the unique number reviewregistry1457. The results of our review are currently under review in a Scopus-indexed journal. This algorithm provides tailored recommendations for prioritizing supporting examinations based on the patient's clinical condition. Diverging from conventional methods, the examinations in the INA-MBD algorithm are not executed sequentially and exhaustively. The diagnostic and management patterns of this algorithm were preliminarily tested in our hospital with a limited sample. Both in theory and practice, the INA-MBD algorithm has proven effective in reducing the time required to diagnose primary malignancy in MBD cases and in lowering costs due to fewer supporting examinations. The main objective is for an early and secure biopsy to speed up the diagnostic process, leading to reduced further examination costs without compromising the success of diagnosing MBD of unknown origin.

**Objective**: The objective of this study was to assess the impact of implementing the INA-MBD algorithm on the direct treatment costs and the time to diagnose primary malignancy in patients with MBD of unknown origin.

## Protocol

### Study design

This research employed a quasi-experimental pre-test and post-test design, categorizing participants into an intervention group and a non-intervention group. The intervention group utilized the INA-MBD algorithm for diagnosing patients with MBD of unknown origin, while the non-intervention group used the intra-hospital conventional diagnosis algorithm.

### Patients and eligibility criteria

In this study, the intervention group comprised consecutively collected patients from inpatients, outpatients, and emergency departments diagnosed with MBD of unknown origin until reaching the required sample size. The INA-MBD algorithm was implemented in the intervention group. For the non-intervention group, medical record data from MBD-diagnosed patients with unknown primary lesions between 2018 and 2022 at RSUP Dr. Sardjito (Yogyakarta) and Dr. Wahidin Sudirohusodo (South Sulawesi) were utilized. We opted for retrospective data for the control group due to the limited number of patients with MBD of unknown origin at our center. The diagnosis of MBD was established using radiological examinations, where the primary tumor was unknown, and there was no previous history of malignancy at presentation. The primary malignancy of MBD was confirmed using histopathological examination.

### Inclusion criteria

a. Patients with a final diagnosis of MBD
b. Patients without a history of malignancy
c. Patients who agreed to participate in the this study and signed the written informed consent form.

### Exclusion criteria

a. Patients with incomplete medical record data
b. Patients who did not undergo operative procedures.

### Study settings

This multicentre research was conducted at two tertiary referral hospital in Indonesia, RSUP Dr. Sardjito in Yogyakarta and Dr. Wahidin Sudirohusodo in South Sulawesi.

### Data collection, management, and analysis

**Data collection**

Patients diagnosed with MBD of unknown origin based on clinical and radiological examinations will be consecutively collected. Those meeting the inclusion and exclusion criteria will be included in the sample. Data will be collected using a standardized case report form for each sample and both intervention and control groups. Patients with incomplete medical records will be excluded.

### Variable

**Independent variable**

**Figure 1.**
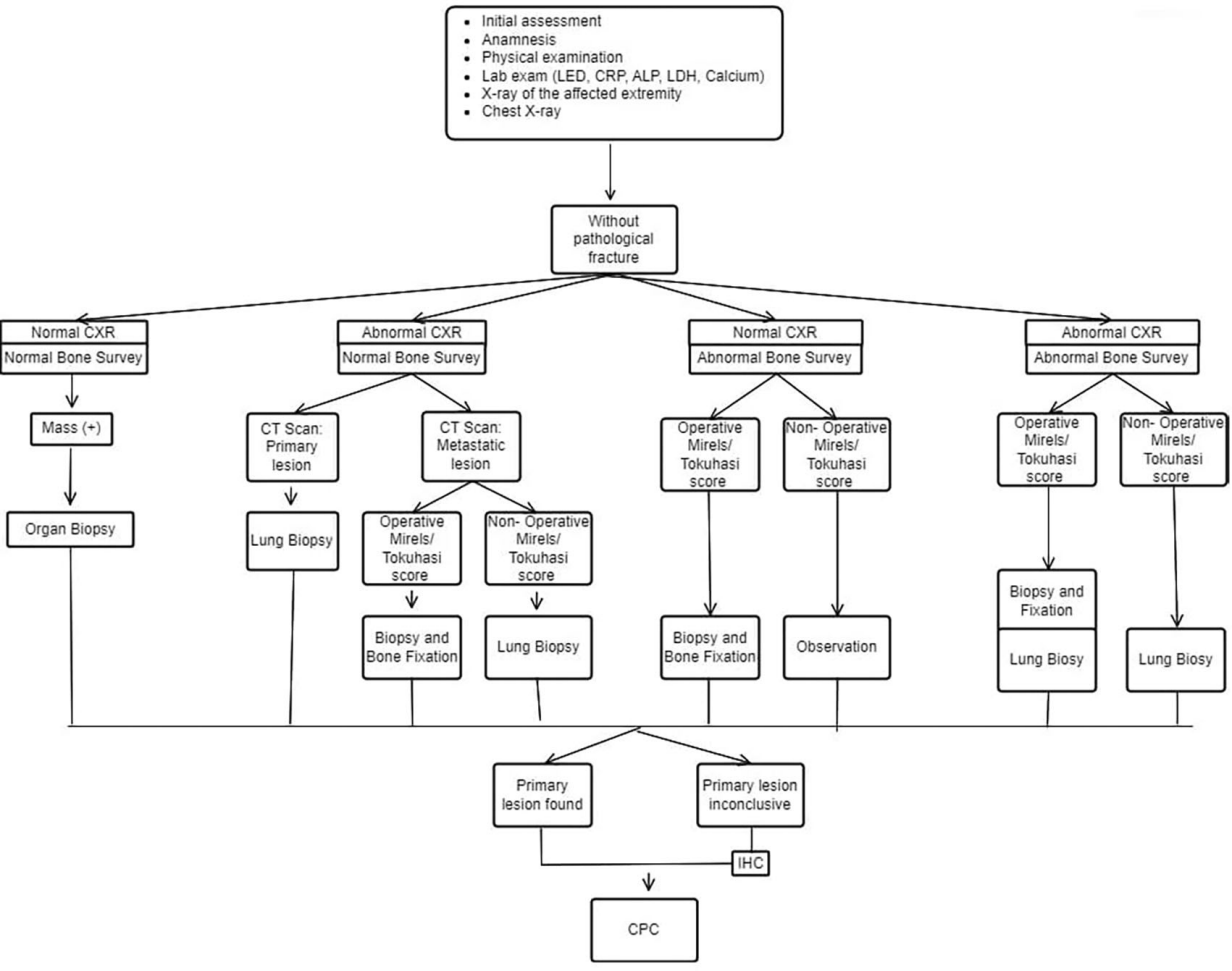
INA-MBD algorithm without pathological fracture. This figure is our original creation synthesized from both literature and our clinical experience. Show steps and list of management in patients with MBD of unknown origin without pathological fracture. CXR (Chest X-Ray), IHC (Immunohistochemistry), CPC (Clinicopathological conference)/ multidisciplinary team discussion

**Figure 2.**
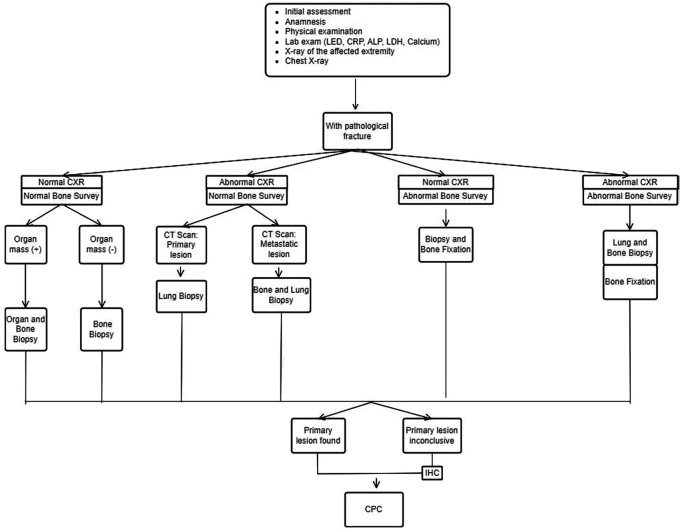
INA-MBD algorithm with pathological fracture. This figure is our original creation synthesized from both literature and our clinical experience. Show steps and list of management in patients with MBD of unknown origin with pathological fracture. CXR (Chest X-Ray), IHC (Immunohistochemistry), CPC (Clinicopathological conference)/multidisciplinary team discussion.

The INA-MBD algorithm ( Figures 1 and 2) will be used as the independent variable. The interventional group will use the INA-MBD algorithm as a management guideline, while the non-interventional group relied on retrospective data from medical records.

### Dependent and confounding variable

The dependent variables were time-to-diagnosis and cost-to-diagnosis. Confounding variables, such as gender, age, type of primary cancer, and metastatic location, were derived from medical records for the non-interventional group and other various sources for the interventional group.

### Data analysis

Bivariate analysis between time to diagnose or cost to diagnose and the use of the INA-MBD algorithm will employ an independent T-test, with Mann-Whitney as an alternative method if the data exhibit abnormal distribution. For the analysis of time to diagnose, the survival rate will be assessed using Kaplan-Meier and log-rank regression tests. To evaluate the hypothesis concerning the cost to diagnose, a covariance test will be employed to analyse the regression mean of both groups. Confounding variables will be controlled using multivariate analysis.

### Research timeline


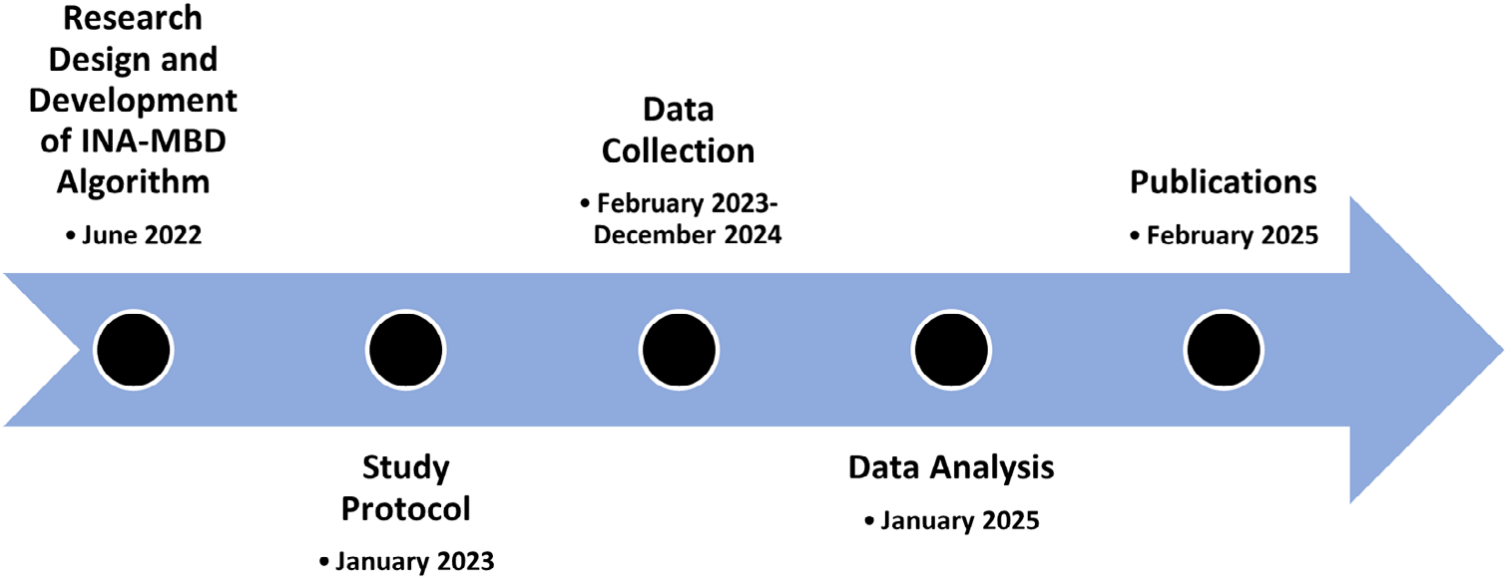


### Dissemination

Upon completion of this study, the findings will be published in a Scopus-indexed journal with a Q2-Q1 quartile ranking. Additionally, the results will be presented at the Continuing Orthopaedic Education meeting held by the Indonesian Orthopaedic Association.

### Study status

The study was in the data collection period and is planned to finish collecting data by December 2024.

### Registration

This research will be registered on Researchregistry.com.

## Discussion

The diagnostic challenge in identifying primary malignancies often leads to patients with MBD of unknown origin being misdiagnosed with primary bone tumors or hematological malignancies. An additional complication is that not all MBD patients have a cancer history, with 71% being diagnosed with their primary malignancy only after a clinical deterioration. ^2^ Another challenge arises when no fractures are found, or patients have no prior history of cancer, making it difficult to diagnose bone metastasis conditions. ^8^ This delay can negatively impact prognosis and increase the risk of Skeletal Related Events (SREs), including pathological fractures or spinal cord compression. ^1^ Therefore, it is crucial to promptly identify the origin of bone metastasis using optimal diagnostic strategies.

Various types and modalities of radiological examinations are recommended for diagnosing MBD of unknown origin. Complaints of pain in patients over 50 years old with multiple osteolytic bone lesions with ill-defined borders, with or without pathological fractures, can suggest MBD condition. Hence, routine chest X-rays or imaging of affected lesions should be performed in patients suspected of having MBD of unknown origin. ^8^^–^^11^ Other radiological examinations that may be conducted include ultrasound, bone scans, Computed Tomography (CT) scans, Magnetic Resonance Imaging (MRI), and PET scans. However, each type of radiological examination has its conditions and limitations.

Katagiri et al. reported that supportive examinations of the gastrointestinal and female reproductive organs often waste time and money, so they are not routinely recommended unless specific symptoms are present. ^10^ Takagi et al. used whole-body CT scans to obtain general body imaging of patients to replace bone scans. ^1^ This study reported a high success rate in diagnosis using a combination of medical history, physical examination, chest X-rays, blood tests, and tumor markers. Lawrenz et al. ^11^ reported that PET/CT scans have poor diagnostic capabilities for MBD of unknown origin, a finding supported by Budak et al., who recommended the use of PET/CT scans only to search for other metastatic lesions, not for diagnosis confirmation. ^12^ Although bone scans are considered the best technique for early diagnosis of bone metastasis, their high cost means they are not routinely performed.

Bone survey can be used as an option to diagnose MBD of unknown origin. The sensitivity of bone surveys in detecting bone lesions ranges only from 44-50%, and it is lower compared to bone scans which have a sensitivity of 78%. However, the detection rate of bone surveys is better than bone scans when accompanied by clinical symptoms suggestive of MBD. The cost of the bone survey is also cheaper, making it still used to evaluate MBD lesions. The use of a bone survey is also recommended to confirm abnormal findings from bone scans. ^13^

In conclusion, when determining the type of supportive examination to use in diagnosing MBD of unknown origin, besides considering the sensitivity and specificity values, the consequences of the examination should also be taken into account. The results of supportive examinations can directly or indirectly affect a person's health condition and outcomes. Health conditions can be influenced by factors such as the accuracy of diagnosis, the success of therapy, or psychological conditions, which are influenced by the cost of diagnostic tests or the amount of management costs given to patients.

Tsukamoto et al. (2021) advocate the diagnostic algorithm for diagnosing MBD of unknown origin. ^14^ Plain X-rays, CT scans, and MRI for all patients with bone lesions. If bone destruction is observed without periosteal reaction, then metastasis is suspected, prompting further examinations like abdominal, thoracic, or pelvic CT scans, tumor markers, serum electrophoresis, Bence Jones protein, and bone scans or PET/CT scans. Biopsy is reserved for the conclusion after completing all imaging and laboratory examinations. ^8^

Tumor marker examinations, including CEA, CA-125, CA19-1, and AFP, exhibit low sensitivity and specificity in determining the primary malignancy in MBD of unknown origin. Tumor markers are not exclusively produced by malignant cells, making serum tumor markers more relevant for therapy monitoring or determining cancer prognosis ^4, 14^ More invasive examinations, such as biopsy, can confirm the origin of the primary malignancy in MBD patients. Biopsy can be performed on metastatic bone lesions, with or without pathological fractures, and on lesions suspected to be primary malignancies. Bone biopsies can confirm the origin of malignancy up to 70%. ^15^

We have devised an algorithm named INA-MBD through clinical and evidence-based methods. The INA-MBD algorithm offers guidelines for managing patients with MBD of unknown origin, in concordance with the patient's clinical condition, whether or not pathological fractures are present. This aims to provide more selective guidance in choosing imaging modalities. The INA-MBD algorithm excludes serum tumor marker examinations as a diagnostic tool for the reasons explained above. It also serves as a guide for performing biopsies not only on suspected primary lesions but also on bone lesions. The measurable variables representing the effectiveness of the INA-MBD algorithm include time-to-diagnosis and the cost-effectiveness of managing patients with MBD of unknown origin. This research aims to establish the INA-MBD algorithm as an evidence-based alternative for managing MBD of unknown origin. The utilization of the INA-MBD algorithm is expected to improve outcomes for patients with MBD of unknown origin and alleviate the financial burden associated with management.

### Ethical considerations

This study has Ethical approval from The Medical and Health Research Ethics Committee (MHREC) Faculty of Medicine, Public Health and Nursing Universitas Gadjah Mada- Dr. Sarjito General Hospital on 09 Jan 2023 and the protocol number is KE/FK/0042/EC/2023 Patients who agreed to participate in the study signed the written consent form.

## Author contributions

Conceptualization: (Y.A.P., A.W., T.A., I., R.M., D.P.) Data Curation: (Y.A.P., D.P., A.W., P.A.S., A.F.) Formal Analysis: (Y.A.P.)., A.W., T.A., I., R.M., D.P) Investigation: (Y.A.P., A.W., P.A.S., A.F) Methodology: (Y.A.P., D.P., A.W., M.P.J., M.A.A.) Software: (Y.A.P., P.A.S., A.F.) Supervision: (T.A., I., RM., D.P., M.P.J., M.A.A.) Validation: (Y.A.P., T.A. I., R.M., D.P., M.P.J., M.A.A.) Writing original draft: (Y.A.P., A.W., P.A.S., A.F.) Writing review & editing: Y.A.P., T.A., I., R.M., D.P., M.P.J., M.A.A., A.W., P.A.S., A.F.).

## Data Availability

No data are associated with this article. Spirit Outcome Checklist is available at *Zenodo: Checklist for” Analysis of the effectiveness and efficiency of the Indonesian metastatic bone disease of unknown origin algorithm (INA-MBD) time to diagnosis and cost to diagnosis: Quasi Experimental Study”*, DOI 10.5281/zenodo.10901731. ^16^ Data are available under the terms of the Creative Commons Attribution 4.0 International license (CC BY 4.0).
