# Supplementary material for: Analysis of the effectiveness and efficiency of the    Indonesian metastatic bone disease of unknown origin algorithm (INA-MBD): time to diagnosis and cost to diagnosis : Quasi-experimental study
Source: F1000Res. 2024 Jul 9;13:333. Originally published 2024 Apr 23. [Version 2] doi: 10.12688/f1000research.146118.2 (PMC11584451; doi:10.12688/f1000research.146118.2)
Supplement: Peer review history

# Reviewer report: Reviewer response for version 2

**DOI:** 10.5256/f1000research.167763.r341851

**Published:** 2024-11-28

**Authors:** Joana Cristo Santos

**Affiliations:** 1 Department of Informatics Engineering, University of Coimbra, Coimbra, Portugal

The article presents a study designed to enhance the effectiveness and efficiency of diagnosing and treating patients with MBD by proposing a pseudo-algorithm and examining the relationships between cost-to-diagnosis and time-to-diagnosis.

 The article is well-structured and offers an innovative approach to improving MBD diagnosis, which could have significant implications for clinical practice. The focus on both cost and time metrics adds practical value to the research, making it highly relevant to healthcare systems aiming to optimize resource allocation.

 However, there are a few areas that could benefit from further elaboration. For instance, the description of the control group lacks detail. Knowing how many patients were included and their demographics would be helpful. Additionally, the methodology would benefit from a clearer explanation of how key variables, such as cost and time, will be measured and analyzed. These details would strengthen the study's credibility and provide a more comprehensive understanding for readers.

Is the study design appropriate for the research question?

Yes

Is the rationale for, and objectives of, the study clearly described?

Yes

Are sufficient details of the methods provided to allow replication by others?

Yes

Are the datasets clearly presented in a useable and accessible format?

Not applicable

Reviewer Expertise:

Meta-Analysis; Oncology Research; Data Science

I confirm that I have read this submission and believe that I have an appropriate level of expertise to confirm that it is of an acceptable scientific standard.

# Reviewer report: Reviewer response for version 2

**DOI:** 10.5256/f1000research.167763.r341849

**Published:** 2024-11-22

**Authors:** Weijie Zhang

**Affiliations:** 1 Department of Orthopaedic Surgery, Zhejiang Provincial Key Laboratory of Cancer Molecular Cell Biology, Life Sciences Institute, the Second Affiliated Hospital, School of Medicine, Zhejiang University, Hangzhou, China

Metastases of unknown primary origin are observed in a subset of cancer patients. Although the primary tumor site can often be identified with advanced diagnostic tools, the process is time-intensive and imposes a considerable financial burden on patients. The current study has the potential to offer direct evidence to assess whether reorganizing clinical practices for these cases could significantly enhance patient survival outcomes or reduce economic strain. The study design is comprehensive, and I fully support the proposed protocol.

Is the study design appropriate for the research question?

Yes

Is the rationale for, and objectives of, the study clearly described?

Yes

Are sufficient details of the methods provided to allow replication by others?

Yes

Are the datasets clearly presented in a useable and accessible format?

Yes

Reviewer Expertise:

Oncology, Molecular Biology, Pathology

I confirm that I have read this submission and believe that I have an appropriate level of expertise to confirm that it is of an acceptable scientific standard.

# Reviewer report: Reviewer response for version 2

**DOI:** 10.5256/f1000research.167763.r300983

**Published:** 2024-07-10

**Authors:** Kevin Christian Tjandra

**Affiliations:** 1 Department of Medicine, Faculty of Medicine, Universitas Diponegoro, Semarang, Central Java, Indonesia

All the revision points have been well resolved

Is the study design appropriate for the research question?

Partly

Is the rationale for, and objectives of, the study clearly described?

Yes

Are sufficient details of the methods provided to allow replication by others?

Partly

Are the datasets clearly presented in a useable and accessible format?

Yes

Reviewer Expertise:

Meta-Analysis, In-Vivo Research, Orthopaedic Research, Oncology Research, Surgery Research, Clinical Research

I confirm that I have read this submission and believe that I have an appropriate level of expertise to confirm that it is of an acceptable scientific standard.

# Reviewer report: Reviewer response for version 1

**DOI:** 10.5256/f1000research.160158.r273174

**Published:** 2024-05-30

**Authors:** Kevin Christian Tjandra

**Affiliations:** 1 Department of Medicine, Faculty of Medicine, Universitas Diponegoro, Semarang, Central Java, Indonesia

Dear Authors,

 Thank you for the opportunity given. This is an interesting in silico research. However, several adjustments are required. To summarize, this research aimed to to enhance the diagnosis of Metastatic Bone Disease (MBD) of unknown origin using the INA-MBD algorithm. Conducted on 128 patients, the quasi-experimental study found that applying the algorithm improved efficiency and reduced costs compared to traditional methods, addressing a high-cost and urgent medical issue.

 In aim to improving the article, listed below are some concerns that were found so maybe editor can consider doing more review on these matters:

1. **Title**

- The title has the clarity and conciseness

1. **Abstract**

 

- Background and purpose: the background abstract is well served.
- Methods: Key improvements include clarifying that patients were randomized to ensure comparability, specifying that the INA-MBD algorithm is the primary diagnostic tool, and detailing how cost analysis was performed. The research also mentioned adjustments for confounding variables and subgroup analyses to increase the robustness of findings. Additionally, reporting baseline characteristics ensured group comparability and notes on follow-up procedures and criteria for evaluating diagnostic accuracy and efficiency were added.

**2.** **Introduction**

  

- The introduction could be improved by incorporating a clearer explanation of the INA-MBD algorithm's development process and evidence supporting its effectiveness. Additionally, providing specific examples or preliminary results demonstrating the algorithm's impact on diagnostic speed and cost reduction would strengthen the introduction. This would help establish the relevance and potential benefits of the algorithm more convincingly.

**3.** **Method**

 To improve the research method, provide a detailed description of the INA-MBD algorithm and how it differs from the conventional diagnosis algorithm, explaining the rationale behind selecting the retrospective data for the control group and ensuring comparability with the intervention group. Include a justification for the sample size calculation, considering the expected effect size and statistical power. Clarify the criteria for patients with a final diagnosis of MBD, specifying diagnostic tests or clinical criteria used, and ensure comprehensive exclusion criteria to avoid confounding variables. Detail how data collection was standardized across different settings and time points, ensuring data quality and completeness, especially for the retrospective control group. Expand on how confounding variables were controlled or adjusted for during the analysis, using multivariate analysis techniques to account for potential confounders. Provide a more detailed plan for the statistical analysis, including assumptions checked for the tests used, handling of missing data, and any sensitivity analyses planned. Include a section on ethical considerations, such as IRB approval and measures to ensure patient confidentiality and data security. Elaborate on the dissemination plan, including specific journals or conferences targeted and any plans for sharing findings with participating hospitals and patients. Provide a detailed timeline of the study phases, including data collection, analysis, and dissemination stages, ensuring alignment with the projected completion date. Confirm the study's registration on Researchregistry.com or provide an update if the registration is complete, including the registration number.

**4.** **Discussion**

 In discussing the research, it's crucial to address various aspects for improvement. Firstly, highlighting specific examples or case studies illustrating misdiagnoses of primary bone tumors or hematological malignancies in MBD patients can elucidate the clinical consequences, including treatment delays and prognosis implications. Additionally, elaborating on the evidence-based diagnostic process for primary malignancy in MBD patients, supported by literature, aids in understanding the rationale behind imaging examination sequences and their limitations. Critically analyzing the use of tumor markers in diagnosing primary malignancies provides insights into their sensitivity, specificity, and relevance in clinical practice, supported by relevant studies. Further, detailing the selective use of supportive imaging examinations, with specific criteria for their application, and comparing the diagnostic accuracy, cost-effectiveness, and time efficiency of different modalities, allows for informed decision-making. Discussion on the role of biopsy in confirming primary malignancy origins, including its accuracy, safety, and implications for treatment, should be incorporated, supported by evidence. Introducing the INA-MBD algorithm requires a comprehensive description of its development process, key components, and how it addresses current diagnostic limitations, emphasizing its potential impact in clinical practice. Clear objectives and hypotheses for evaluating the INA-MBD algorithm, along with the methodology for assessing its effectiveness and efficiency, are essential. Additionally, specifying outcome measures, such as time-to-diagnosis and cost-effectiveness, and addressing potential confounding variables in the analysis are crucial. Lastly, discussing the research's implications on clinical practice and healthcare policy, offering recommendations for INA-MBD algorithm implementation, and suggesting areas for future research would provide a comprehensive outlook.

**5.** **Conclusion**

- The conclusion is well-executed

**6.** **Additional Information**

- The additional information provides sufficient information

I hope the suggestions above will improve the article to be indexed. Once again, thank you for the opportunity given.

Is the study design appropriate for the research question?

Partly

Is the rationale for, and objectives of, the study clearly described?

Yes

Are sufficient details of the methods provided to allow replication by others?

Partly

Are the datasets clearly presented in a useable and accessible format?

Yes

Reviewer Expertise:

Meta-Analysis, In-Vivo Research, Orthopaedic Research, Oncology Research, Surgery Research, Clinical Research

I confirm that I have read this submission and believe that I have an appropriate level of expertise to confirm that it is of an acceptable scientific standard, however I have significant reservations, as outlined above.

# Response

**Published:** 2024-06-10

**Authors:** Yuni Artha Prabowo Putro

**Affiliations:** Orthopaedics and Traumatology, Gadjah Mada University, Yogyakarta, Special Region of Yogyakarta, Indonesia

Thank you for your kind responses and suggestions. We have adjusted and improved several parts of our manuscript based on the reviewer's comments.

 Abstract

- Methods: This research will be conducted using a quasi-experimental design, meaning the sample will receive the intervention without randomization. We chose this design due to the small number of patients with metastatic bone disease of unknown origin, as indicated by both the literature and our clinical experience. Therefore, we will use retrospective data from electronic medical records for the control group, which did not use INA-MBD as a management algorithm, and prospective data for the intervention group, which will use INA-MBD. This decision was made to ensure we have a sufficient sample size.
- To assess the cost-effectiveness of the INA-MBD algorithm, we only included and analyzed the costs based on ICD.9 coding. This approach was chosen to minimize rate variations between the two hospitals where our research was conducted.
- The baseline characteristics of the data samples will be presented descriptively. The data includes initials, age, initial diagnosis, details of supporting examinations, and categorization into control or intervention groups. The final diagnosis will be recorded as the primary malignancy if identified, or as MBD of unknown origin if not identified. The diagnosis of primary malignancy or MBD is based on the histopathological result.

Introduction

- The INA-MBD algorithm was developed through a rigorous scientific process. We conducted a systematic review, registered in the research registry with the unique number reviewregistry1457. The results of our review are currently under review in a Scopus-indexed journal.
- The diagnostic and management patterns of this algorithm were preliminarily tested in our hospital with a limited sample. Both in theory and practice, the INA-MBD algorithm has proven effective in reducing the time required to diagnose primary malignancy in MBD cases and in lowering costs due to fewer supporting examinations.

Method

- Development of INA-MBD Algorithm 

The INA-MBD algorithm is a management protocol for MBD of unknown origin, developed based on systematic reviews and our clinical experience treating MBD patients at a tertiary referral hospital. INA-MBD differs from other MBD management algorithms because it tailors subsequent examinations based on whether the patient has experienced a pathological fracture. Additionally, we excluded tumor marker testing from the INA-MBD algorithm due to the low level of evidence supporting their use in diagnosing primary malignancies in MBD cases. Our algorithm incorporates a clinicopathological conference (CPC) as a diagnostic tool.

 The widely used algorithm by Tsukamoto et al. (Curr. Oncol. 2021, 28(5), 3347-3372; https://doi.org/10.3390/curroncol28050290) significantly differs from the INA-MBD algorithm we propose.

- Rationale to use retrospective data

We used retrospective data for the control group due to the limited number of patients with MBD of unknown origin at our center. Therefore, we employed retrospective data for the control group (where the INA-MBD algorithm had not been applied) and prospective data for the intervention group (where the INA-MBD algorithm was applied).

- Sample size

To determine the sample size, we used the formula for the difference in continuous data between the two groups. With a 95% confidence interval and 80% power, and based on the study by Kitagawa et al. (2018), the time required to diagnose MBD of unknown origin was 16 weeks. The researchers considered a meaningful difference to be 8 weeks. Thus, the minimum sample size needed was 63 per group or 126 in total.

- Final diagnosis of MBD definition

In diagnosing cases of MBD, we used radiological examinations to identify bone lesions where the primary tumor was unknown and there was no previous history of malignancy at presentation. The primary malignancy of MBD was confirmed using the histopathological examination.

- Data collection

Patients diagnosed with MBD of unknown origin based on clinical and radiological examinations will be consecutively collected. Those meeting the inclusion and exclusion criteria will be included in the sample. Data will be collected using a standardized case report form (Supplementary Material) for each sample and both intervention and control groups. Patients with incomplete medical records will be excluded.

- IRB approval

This study has been approved by the Medical and Health Research Ethics Committee (MHREC) of the Faculty of Medicine, Public Health, and Nursing at Universitas Gadjah Mada – Dr. Sardjito General Hospital, with reference number KE/FK/0162/EC/2024.

- Dissemination plan

Upon completion of this study, the findings will be published in a Scopus-indexed journal with a Q2-Q1 quartile ranking. Additionally, the results will be presented at the Continuing Orthopaedic Education meeting held by the Indonesian Orthopaedic Association

 We have revised our latest manuscript to incorporate the comments and suggestions from the reviewers.
